# The impact of prices on alcoholic beverage consumption in Chile

**DOI:** 10.1371/journal.pone.0205932

**Published:** 2018-10-22

**Authors:** Daniel Araya, Guillermo Paraje

**Affiliations:** 1 Universidad Adolfo Ibáñez, Santiago, Chile; 2 Business School, Universidad Adolfo Ibáñez, Santiago, Chile; RTI International, UNITED STATES

## Abstract

**Introduction and objective:**

Chile is among the countries with the highest alcohol consumption per capita in Latin America, but little has been done in the way of public policy and policy research to overcome this problem. The objective of the present study is to estimate demand elasticities (own-price, cross-price, expenditure and quality) for three groups of alcoholic beverages in Chile: spirits, wines, and beers.

**Data and methods:**

The study uses data from the VII Encuesta de Presupuestos Familiares (Family Budget Survey) 2011–2012 conducted by the National Institute of Statistics. Because of problems with the quality of the measurement units, hot-deck imputation method was used with the alcohol purchases that presented problems. To estimate the demand elasticities, the Almost Ideal Demand System (AIDS) method proposed by Deaton was used. Quality decisions were estimated for each beverage separately using an equation proposed by Deaton in the three-step AIDS.

**Results:**

The estimated elasticities were more inelastic for spirits (-0.14, P<0.01), followed by wines (-0.77, P<0.01) and beers (-0.93, P<0.01). Spirits reported less sensitivity to changes in the total budget, while wines reported the most sensitivity to changes in the total budget (expenditure elasticity). Wines also reported the most sensitivity related to quality for changes in the total budget (0,20, meaning that a 10% increase in a household’s total expenditure increases the “quality” of purchased wines by 2%).

**Conclusions:**

Own-price elasticities reported for spirits, wines and beers are consistently negative, and inelastic, in line with international evidence. Although own-price elasticities for spirits is more inelastic than found in most studies, its quality-elasticity is more positive and greater. This could be explained by the greater price dispersion of spirits, as well as spirits (and wine) being consumed more than beers in Chile. This in turn may be because consumers have the option of switching to cheaper, Chilean-produced spirits such as pisco and wines when prices rise. The existence of these relatively broad quality-elasticities points to the need for a change in the alcohol tax structure from the current ad-valorem tax to a specific tax that could reduce price dispersion and curb total consumption.

## Introduction

Harmful use of alcohol is related to over 200 diseases [1]. In 2010 alcohol consumption was the third greatest risk factor related to loss of healthy life years in the world (5.5%) [2]. Increasing alcohol taxes has been highlighted as one of the most cost-effective ways to reduce harmful use of alcohol [3–6].

Knowledge of the characteristics of the demand for alcoholic beverages is fundamental to understanding the potential impact of a tax increase. One of the fundamental parameters of alcohol demand is its price elasticity, which is, ceteris paribus, the relative impact of a price increase on the amount consumed (i.e. the percentage of the decline in demand if the price increases by 10%). In addition, it is important to know the income elasticity of demand, which reveals, ceteris paribus, how demand responds to an increase in consumers' incomes. If demand for a good increase with an increase in income (i.e., income elasticity is positive), the good is shown to be normal, while if demand declines with an increase in consumer revenue, then it is an inferior good. When expenditure data is available, an expenditure elasticity can be estimated, with the same interpretation as the income elasticity (ie, a normal good is one with a positive expenditure elasticity). Even when both income and expenditure data are at hand, it is recommended to use expenditure as a proxy for material affluence, as expenditure is less susceptible to measurement errors than income [7].

There is extensive literature for different countries on the impact of prices on the consumption of alcoholic beverages. A meta-analysis that included 112 estimates for alcoholic beverages in different countries (mainly developed nations) reported mean price elasticities for alcohol (all varieties), beers, wines and distilled spirits of -0.51, -0.46, -0.69 and -0.8 respectively [8]. Another meta-analysis estimated that, compared to all other alcoholic beverages, beers are the most inelastic, while no significant difference in elasticity was found between wines, distilled spirits and other alcohol [9]. A review of 12 estimates for low- and middle-income countries (excluding Latin American countries) reported elasticities' of -0.64 for all alcohols, -0.5 for beers and -0.79 for other alcoholic beverages [10].

There is at least one estimate of elasticities for alcoholic beverages for Latin America, a study on demand for wine in Chile [11]. This work seeks to demonstrate a structural break in the demand for wines in Chile using time series. The estimated price elasticities were -0.076 for the period 1949–1982 and -0.48 for the period 1983–1998; while income elasticity was -0.304 in the former period and -1.724 for the latter. The negative values for income elasticities are counter-intuitive, as they would imply wine is an inferior good, which is at odds with international evidence [9]. This oddity may be related to a misspecification in the estimated model, though we do not have the ability to properly assess this, as we do not have the data used to estimate the model.

The case of Chile is of particular interest, given that it has the highest annual per capita consumption of pure alcohol in Latin America, 9.6 litres of pure alcohol per population over the age of 15 in 2008–2010 [1]. If this consumption is adjusted for the drinking population, it ascends to 14.63 litres per year [1]. This pattern of consumption is far from balanced, as it is concentrated in just 1.6 days a week [12], with frequent episodes of harmful and dangerous consumption. In 2016 51.1% of alcoholic beverage consumers had a recent episode of excessive consumption (during the previous month). The percentage rises to 57.7% for drinkers between 12 and 18 years old and to 60.1% for drinkers between 19 and 25 years old [13]. The monthly prevalence of alcohol consumption has remained practically unchanged since 1996, reaching 46% in 2016. Adults between 26 and 34 years of age are those with the highest drinking prevalence, 58.1%, while those belonging to high income groups have the highest prevalence: 51.3% [13].

In Chile, alcoholic beverages are currently taxed at an ad valorem rate of 31.5% for distilled spirits and liquors and 20.5% for beers, wines and sparkling wines. The tax base for the tax is the same tax base as the VAT and is applied at the last stage of commercialization. These taxes were increased in 2014 from 27% for distilled spirits and liquors and 15% for beers, wines and sparkling wines. This ad-valorem tax is not optimal to discourage consumption of beverages with high-alcohol content. For example, wine is taxed at the same rate as beer, even though wine may have three times more alcohol per litre than beer. This also increases the price dispersion within each category of alcoholic beverages, incentivizing the substitution towards cheaper brands (down-trading).

The sale of alcohol to minors and its consumption in public spaces is forbidden in Chile. However, there is no legal restriction on advertising, promotion or sponsorship by producers of alcohol, nor is there any sort of legislation implementing minimum prices or sales restrictions related to prices, measures that have been proven to be effective at discouraging consumption [5].

The purpose of this study is to estimate the price elasticity of demand for wines, beers and spirits in Chile, with the goal of providing input for further taxation policies on alcohol consumption in Chile and the region.

## Methodology and data

Demand elasticities were obtained using the Almost Ideal Demand System (AIDS) [14] modified to adjust for household preferences by demographic characteristics [15, 16]. Elasticities were estimated for the full sample and for specific socio-demographic groups, categorized by area of residence, sex, and education of the household head. The AIDS, adjusted by demographic characteristics, models the households’ spending decision following the Eq (A):

$$w_{i}=\alpha_{i}+\sum_{j=1}^{k}\gamma_{ij}\;\ln\;P_{j}+{(\beta }_{i}+\eta_{i}z)ln\left\{\frac{X}{{\bar{m}}_{0}(z)P}\right\}$$ (A)

Where X is the total expenditure on the k goods analyzed, *w*_*i*_ is the budget share of the good i in the expenditure X (*w*_*i*_ = *P*_*i*_*Q*_*i*_/*X*), *P*_*j*_ is the price of the good j (unit value in this case) and *P* is a price index. The component *z* is a vector of socioeconomic variables including the natural logarithm of the number of people in the household; the proportion of people over 15 years in the household; the proportion of women over the age of 15 years in the household; and the sex, age, and educational level of the head of household (primary incomplete, secondary incomplete, secondary complete, and some university education). The function ${\bar{m}}_{0}(z)$ corresponds to the demographic component of the expenditure scaling function *m*_0_(*z*,*p*,*u*) which scales the expenditure of the household by its demographic characteristics [16].

All equations for the k goods are estimated simultaneously by a Seemingly Unrelated Regression (SUR). This means the households are choosing at the same time how much of their total expenditure is allocated to each of the k goods.

The price index is defined as in Eq (B):

$$\ln P=\alpha_{0}+\sum_{i=1}^{k}\alpha_{i}\;\ln P_{i}+\frac{1}{2}\sum_{i=1}^{k}\sum_{j=1}^{k}\gamma_{ij}\;\ln\;P_{i}\;\ln P_{j}$$ (B)

Replacing Eq (B) in Eq (A) one obtains an equation which models the household’s decision which can be estimated by maximum likelihood:

$$\begin{array}{l} w_{i}=(\alpha_{i}-\beta_{i}\alpha_{0})+\sum_{j=1}^{k}\gamma_{ij}\;\ln P_{j}\\ \;+(\beta_{i}+\eta_{i}^{\prime }z)\left\{\ln\frac{X}{{\bar{m}}_{0}(z)}-\sum_{i=1}^{k}\alpha_{i}\;\ln P_{i}-\frac{1}{2}\sum_{i=1}^{k}\sum_{j=1}^{k}\gamma_{ij}\;\ln P_{i}\;\ln P_{j}\right\} \end{array}$$ (C)

As we do not have any data on actual prices of purchased goods, we proxy these prices with unit values (ie, the ratio between expenditures and physical quantities).

It can be demonstrated that the price/cross-price and expenditure elasticities are as shown by Table 1 [15]:

**Table 1:** AIDS Elasticities.

Expenditure elasticity	$\mu_{i}=1+\frac{\left(\beta_{i}+\eta_{i}^{\prime }z\right)}{w_{i}}$
Non-compensated own and cross price elasticity	$\epsilon_{ij}^{u}=-\delta_{ij}+\frac{1}{w_{i}}\left(\gamma_{ij}-[\beta_{i}+\eta_{i}^{\prime }z]\times [\alpha_{j}+\sum_{i=1}^{k}\gamma_{ij\;}\ln p_{i}]\right)$
Compensated own and cross price elasticity*	$\epsilon_{ij}^{C}=\epsilon_{ij}^{u}+\mu_{i}w_{j}$

* Compensated elasticities obtained by a Slutsky equation.

Where *δ*_*ij*_ is a Kronecker delta, which is equal to 1 if *i* = *j* and zero otherwise.

In addition, following the method proposed by Deaton [7, 17], a quality decision was estimated in order to understand the nature of the quality (measured as unit value) decision of the households. The decision estimated corresponds to the following equation:

$$lnv_{Gic}=\alpha_{G}+\beta_{G}lnx_{ic}+\gamma_{G}z_{ic}+\sum_{H=1}^{N}\psi_{GH}lnp_{Hc}+u_{Gic}$$

Where *lnv*_*Gic*_ is the natural logarithm of the unit value of the good *G*, chosen by the household *i*, from the cluster *c* (census units). *x*_*ic*_ is the total expenditure of the household; *z*_*ic*_ a vector of socioeconomic variables, which are the same ones used for the AIDS model; *p*_*Hc*_ is a vector of the unobserved price vector of the good *H* (spirits, wines, and beers); and *u*_*Gic*_ is an idiosyncratic error. Assuming that households of the same cluster observe the same prices, the equation is estimated by cluster fixed effects, solving the problem of the unobserved price vectors. Once the equation is estimated, the coefficient *β*_*G*_ can be interpreted as the quality elasticity of the total expenditure, defined as the proportional change in unit values as a result of a change of total household expenditure (eg, a 1% change).

The estimates are obtained using the quaids command in Stata 14 and standard errors are calculated by a clustered Bootstrap of 1000 repetitions.

The database used corresponds to the household expenditure survey VII Encuesta de Presupuestos Familiares (Family Budget Survey) (VIIEPF) that the Chilean National Statistics Institute implemented between November 2011 and October 2012. The data covers 10,501 households, of which 3,952 reported expenditure on alcohol. Households participating in the survey had to record three variables related to their purchases (not only for alcohol, but for most goods): expenditure, quantities or “portions” (in physical units) and unit of measurement of those quantities or “portions”. Households could report more than one purchase of alcohol and, thus, the total number of observations of purchases of alcoholic beverages is 8,511. It is worth noting that only off-premise purchases (eg, from a supermarket, kiosk, or store) are considered. We explicitly chose not to consider on-premises purchases (eg, in restaurants, bars, or pubs), as such purchases relate not only to the alcoholic beverage but many other services (eg, being served at a table, the location and facilities of the premises). Thus, we could not estimate how much of the on-premise purchases correspond solely to the value of alcoholic beverages. In any case, off-premise consumption represents close to 85% of the total volume of alcohol consumption in Chile [18].

The sample is representative on a national level and at the level of the Santiago Metropolitan Region. The smallest geographic unit corresponds to census unit (with 8 households on average), which is used as a cluster for estimating quality elasticities of total expenditures. The sample includes 1,265 census units, of which 1,133 contain at least one household that consumes alcohol (89.57% of the sample).

As in most expenditure surveys, there are measurement errors in variables regarding purchases. It is not possible to identify such errors in monetary expenditures or in the number of purchased units. However, measurement errors in units of measurement are clear. While some households recorded their purchases in comparable units (ml, litres, cubic centimetres), others only reported buying “portions” (“unidades” in Spanish). These observations are, clearly, measurement errors. For a household reporting a purchase of 2 litres of beer for a total of CLP 3,000, we can estimate a unit value of CLP 1,500 per litre. For a household reporting a purchase of 2 “portions” of beer we cannot estimate a unit value, as we do not know what those “portions” are.

Table 2 shows that 34.6% of purchases on alcoholic beverages incorrectly register the unit of measurement of alcoholic beverages purchased, making it impossible to calculate the unit values of the purchases, even when monetary expenditures and number of units are correctly reported (at least in appearance).

**Table 2:** Percentage and total number of observations (purchases) of alcoholic beverages by expenditure decile.

Decile	Spirits		Wines		Beers		All Beverages	
	% Missings	Total number of observations	% Missings	Total number of observations	% Missings	Total number of observations	% Missings	Total number of observations
1	28.6%	35	21.5%	158	42.3%	189	32.5%	382
2	33.8%	74	23.8%	168	33.8%	266	30.5%	508
3	38.8%	80	28.1%	228	40.7%	297	35.7%	605
4	35.5%	124	19.4%	279	37.4%	353	30.4%	756
5	38.3%	128	20.8%	274	36.1%	363	31.0%	765
6	36.1%	166	23.3%	292	38.3%	444	33.0%	902
7	43.9%	223	29.8%	373	38.0%	471	36.4%	1,067
8	38.1%	294	26.1%	380	41.2%	420	35.1%	1,094
9	40.3%	278	31.6%	437	36.9%	483	35.7%	1,198
10	45.7%	376	35.4%	413	37.8%	445	39.4%	1,234
Total number of observations	40.1%	1,778	27.0%	3,002	38.1%	3,731	34.6%	8,511

Source: Own construction from VIIIEPF

Note: Each observation is a purchase of alcohol by a household. Households may have several purchases of alcoholic beverages.

To resolve this problem, an imputation of wrongly registered purchases was performed using the hot-deck method [19]. This method separates households into two groups: one with a well-registered measurement unit (donors) and a recipient group with *missing* values. Strata are then created for both groups according to variables that could be related to the missing pattern, in this case, deciles of total household spending. Lastly, by decile of total expenditure, each observation with missing values in the measurement unit is randomly imputed with replacement of a selected observation from the donor group. What is imputed is the unit of measurement of the purchase, as the rest of the information (eg, expenditure value and quantity) is recorded correctly. The advantage of this non-parametric method over parametric methods is that it does not reduce the variance of the observations, which is key to estimating parameters like elasticities [20].

A series of requirements must be fulfilled for the hot-deck method to produce consistent and unbiased estimates. First, the missing values in each cell must be randomly distributed so that, when the data is imputed, the distribution is replicated in the donor cell. Secondly, the variables chosen to create the cells must have some theoretical relationship with the value of the variable with omissions.

In this case, the decile of total per capita household spending is taken as the stratified variable. This is because there is a positive relationship between the proportion of observations with measurement errors and the average total per capita spending per decile. The partial correlation coefficients between total average spending per decile and the number of missing values in distilled spirits, wines and beers are 0.94 (p<0.01), 0.88 (p<0.01) and 0.62 (p<0.1), respectively. Variables that might be related to the proportion of missing values, such as the proportion of women household heads, do not show any statistical relationship to such a proportion and, thus, are not considered for stratification. Other variables such as educational level or household size are strongly correlated to total expenditure, which means there is no practical gain in considering them as stratifiers.

Once the hot-deck imputation was performed, an analysis to eliminate possible outliers generated by the imputation was conducted. Firstly, households with budget shares, quantities or unit values for each alcoholic drink lower/higher than 3 standard deviations from the mean values were eliminated from the analyses (227 households or 2.2% of the total). Secondly, sensitivity analyses were conducted using 4 and 5 standard deviations as thresholds. No relevant differences were found considering the different thresholds, and, thus, only results from the first threshold (3 standard deviations) are presented. Outliers were eliminated for both donor and recipient households.

Table 3 shows the average values and standard deviations of the demographic variables used in the estimation, disaggregated by total expenditure quintile. It can be seen that 40.65% of heads of household are women and that this percentage is higher among households with lower total expenditure. Furthermore, the average age of household heads is 52 years, higher in households in the lower quintile. The average household size is larger in those with higher expenditure, as is the percentage of people over 15 years of age and of women over 15 years.

**Table 3:** Mean and standard deviation of used variables^1^.

Variable	Quintile 1		Quintile 2		Quintile 3		Quintile 4		Quintile 5		Total	
	Mean	Standard Deviation	Mean	Standard Deviation	Mean	Standard Deviation	Mean	Standard Deviation	Mean	Standard Deviation	Mean	Standard Deviation
% of women head of household	55.4%	0.50	45.2%	0.50	40.8%	0.49	35.1%	0.48	26.8%	0.44	40.6%	0.49
Age of head of household	56.9	16.9	53.1	16.0	51.4	15.1	49.6	14.7	49.2	13.3	52.1	15.5
Household monthly total expenditure^2^	$162,354	58,392	$338,622	47,186	$537,031	68,922	$866,517	129,983	$2,104,374	1,100,673	$801,738	852,407
Number of people per household	2.6	1.52	3.4	1.57	3.7	1.71	3.8	1.80	3.9	1.7	3.5	1.73
% of people older than 15 years	85.7%	0.22	81.5%	0.21	82.3%	0.20	81.5%	0.20	81.8%	0.20	82.5%	0.21
% of women older than 15 years	51.0%	0.31	44.6%	0.24	45.8%	0.23	43.5%	0.22	42.4%	0.22	45.5%	0.25
% of heads of household without education or primary incomplete	31.4%	0.46	20.4%	0.40	12.2%	0.33	7.8%	0.27	2.4%	0.15	14.9%	0.36
% of heads of household with secondary incomplete	33.9%	0.47	32.9%	0.47	30.0%	0.46	20.1%	0.40	7.9%	0.27	24.9%	0.43
% of heads of household with complete secondary	29.5%	0.46	35.8%	0.48	38.9%	0.49	35.8%	0.48	20.4%	0.40	32.0%	0.47
% of heads of household with some university studies	5.2%	0.22	10.9%	0.31	18.9%	0.39	36.4%	0.48	69.4%	0.46	28.2%	0.45

Source: Own construction from VIIIEPF

^1^: Mean in the upper value, standard deviation in the lower value

^2^: In Chilean pesos.

There is significant disparity between quintiles in terms of the average level of education attained by heads of households. 31.4% of household in the quintile with the lowest total expenditure have no formal schooling or did not complete primary school, while just 5.2% have university qualifications of some kind. In the quintile with the highest expenditure on alcohol, just 2.4% of household heads have no formal education or did not finish primary school, while 69.4% have some sort of university qualification.

Table 4 shows alcoholic beverage consumption by type of beverage and expenditure quintile. 10.3% of households in the total sample reported spending on distilled spirits, 19.7% spent on wines and 23.1% on beers. In all beverage groups, the percentage of households with expenditure on alcohol increases significantly in relation to the quintile of total expenditure. The average monthly quantity consumed is 3.5 litres of spirits, 4.8 litres of wine and 13.6 litres of beer, which increases by quintile. Mean unit values and total expenditure in Chilean pesos also increase by quintile for all types of beverages, except for the first quintile of beer consumption, which shows the second highest unit value.

**Table 4:** Quantity, expenditure, budget share, unit value and percentage of households with reported spirit expenditure by quintile con total expenditure.

Variable	Quintile 1		Quintile 2		Quintile 3		Quintile 4		Quintile 5		Total	
	Mean	Standard Deviation	Mean	Standard Deviation	Mean	Standard Deviation	Mean	Standard Deviation	Mean	Standard Deviation	Mean	Standard Deviation
**Spirits**												
Average quantity consumed per month^1^	1,710	464	2,366	1,554	2,135	1,148	2,759	1,812	4,508	19,957	3,485	14,143
Average expenditure in spirits per month^2^	$5,797	2,199.0	$8,713	4,625.2	$8,440	5,497.1	$13,592	10,331.3	$15,683	14,143.2	$13,373	11,962.6
Budget share	3.0%	0.01	2.5%	0.01	1.6%	0.01	1.6%	0.01	0.8%	0.01	1.3%	0.01
Average unit value^2^	$3.48	1.2	$4.13	1.9	$4.15	2.3	$5.02	2.3	$5.12	2.6	$4.84	2.43
% of households with spirit expenditure	1.3%	0.11	3.7%	0.19	7.2%	0.26	13.8%	0.34	25.7%	0.44	10.3%	0.30
**Wines**												
Average monthly quantity consumed^1^	2,986	1,791	3,668	2,678	4,312	4,964	4,565	4,330	6,037	18,021	4,789	11,082
Average expenditure in wines per month^2^	$4,883	2,317.9	$6,338	4,119.4	$7,515	5,865.5	$9,591	8,580.9	$18,075	22,474.8	$11,292	15,021.3
Budget share	3.0%	0.01	1.9%	0.01	1.4%	0.01	1.1%	0.01	0.9%	0.01	1.3%	0.01
Average unit value^2^	$1.80	0.7	$1.92	0.8	$2.10	1.2	$2.37	1.1	$3.35	1.6	$2.55	1.41
% of households with wine expenditure	6.3%	0.24	13.8%	0.35	20.2%	0.40	24.6%	0.43	33.7%	0.47	19.7%	0.40
**Beers**												
Average monthly quantity consumed^1^	5,473	6,785	7,900	9,004	12,581	17,104	16,085	19,515	16,323	20,772	13,614	18,121
Average expenditure in beers per month^2^	$3,937	2,451.3	$6,003	4,442.6	$7,613	6,530.8	$10,251	8,445.1	$12,373	11,747.5	$9,375	9,078.5
Budget share	2.3%	0.02	1.8%	0.01	1.4%	0.01	1.2%	0.01	0.7%	0.01	1.2%	0.01
Average unit value^2^	$1.18	0.9	$1.05	0.6	$1.08	0.7	$1.11	0.7	$1.26	0.8	$1.15	0.77
% of households with beer expenditure	6.9%	0.25	16.5%	0.37	24.6%	0.43	30.4%	0.46	37.2%	0.48	23.1%	0.42

Source: Own construction from VIIIEPF

^1^: In millilitres

^2^: In Chilean pesos.

**Fig 1:**
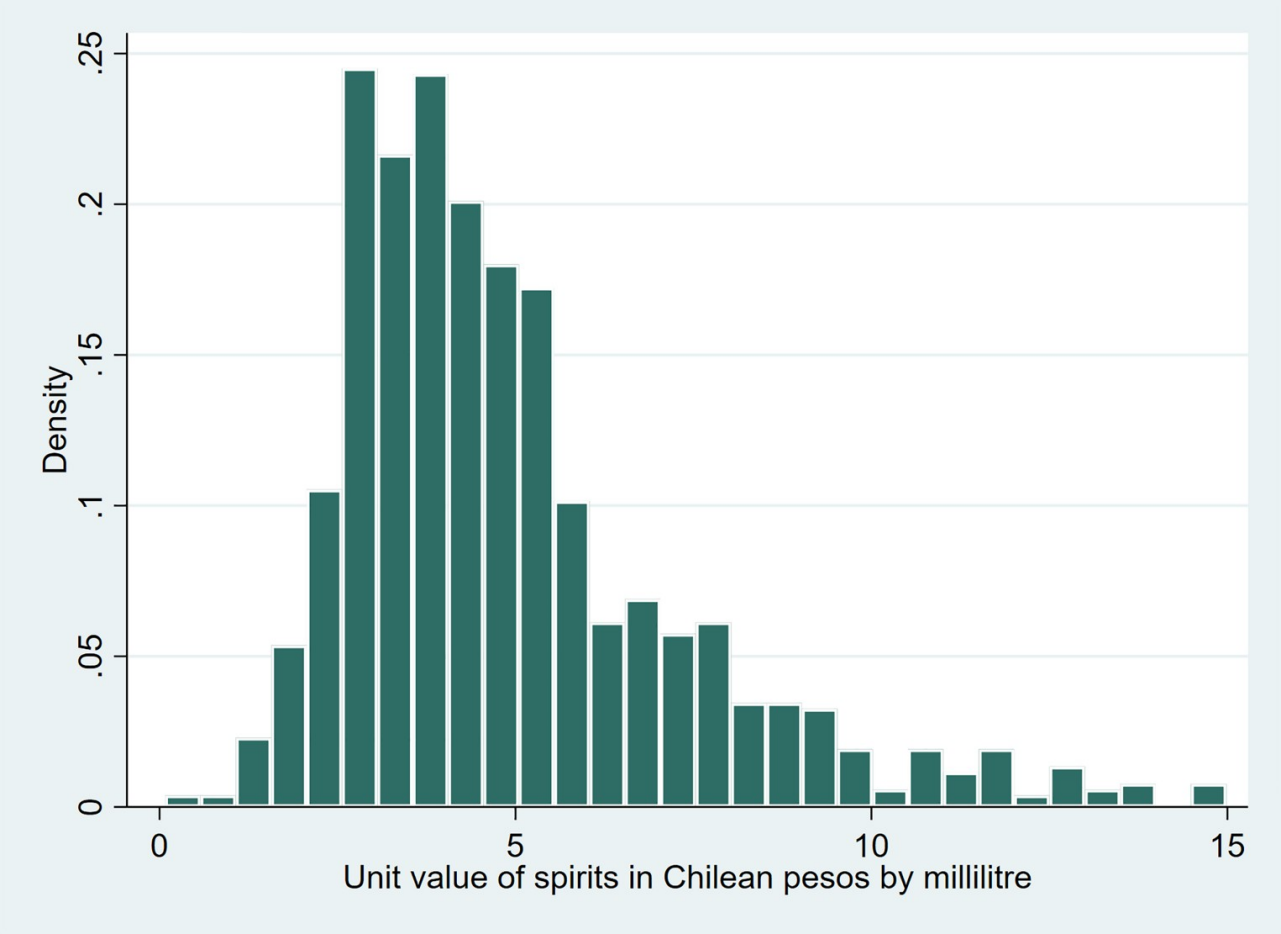
Histogram of unit values of spirits.

**Fig 2:**
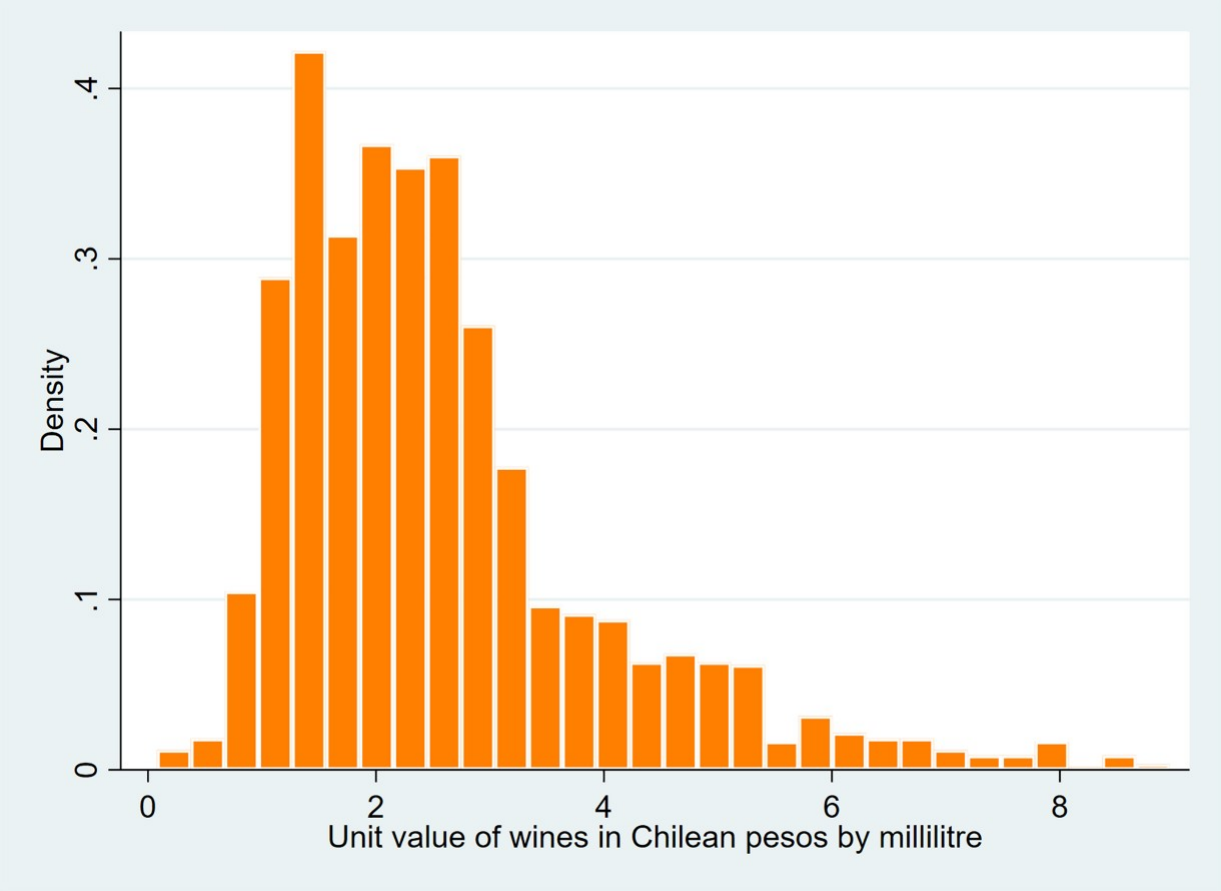
Histogram of unit values of wines.

**Fig 3:**
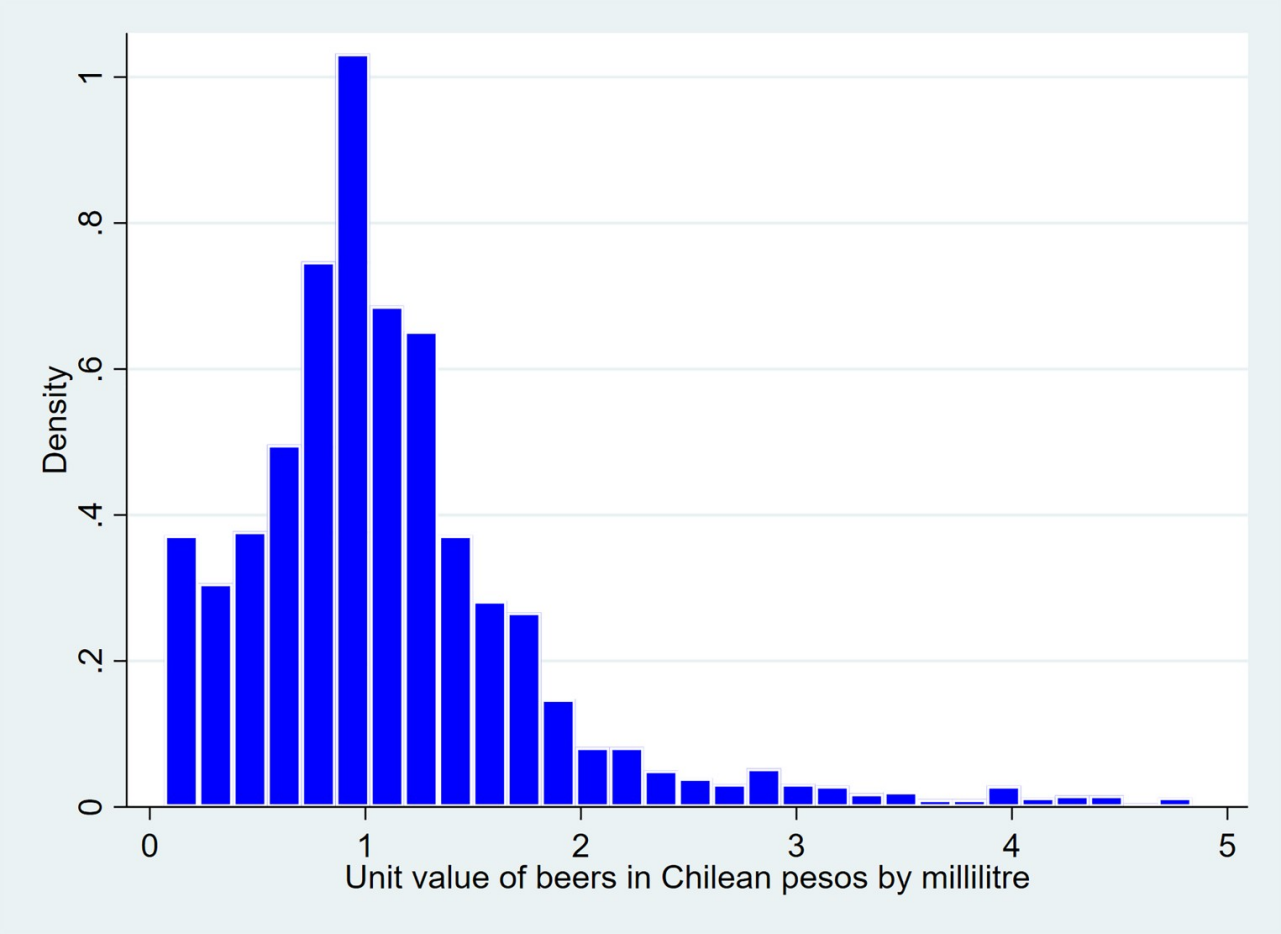
Histogram of unit values of beers.

Figs 1 to 3 show dispersions of unit values per type of beverage. As can be observed, the dispersion of such values can be high for some beverages (eg, spirits). This is due not only to the large variety of drinks in some of the categories (eg, spirits include beverages like pisco, rum, vodka, whisky), but also possibly to the alcohol tax structure. It is well-researched that ad-valorem tax structures tend to increase the price dispersion of products, unlike specific excises, which produce the opposite effect [21].

Figs 1–3 also show that, on average, spirits are the most expensive alcoholic beverage by unit value, followed by wines and then beers.

## Results

Estimated elasticities are reported in Table 5. These show that estimated own price elasticities for distilled spirits, wines, and beers are equal to -0.14, -0.77 and -0.93, respectively. Only the elasticity of spirits is not significant at 1%, showing a significance at 10%. This means that, ceteris paribus, an increase of 10% in the price of distilled spirits would reduce average household consumption by 1.4%. In the case of beer consumption, however, the reduction would be 9.3% and the reduction in wine consumption would be 7.7%.

**Table 5:** Cross Price, expenditure, and quality-expenditure elasticities matrix^1^.

	Spirits		Wines		Beers	
**Spirits**	-0,143	*	-0,494	***	-0,070	
	0,078		0,089		0,052	
**Wines**	-0,342	***	-0,770	***	-0,050	
	0,057		0,092		0,055	
**Beers**	-0,053		0,002		-0,929	***
	0,083		0,076		0,065	
**Expenditure Elasticity**	0,707	***	1,161	***	0,980	***
	0,094		0,101		0,103	
**Quality Expenditure Elasticity**	0,151	***	0,197	***	0,117	***
	0,003		0,002		0,002	

*: Significant for significance levels greater than or equal to 10%

**: Significant for significance levels greater than or equal to 5%

***: Significant for significance levels greater than or equal to 1%

^1^: Upper value corresponds to elasticities. Lower value corresponds to standard deviation

Total expenditure elasticity is also lower for distilled spirits, at 0.71, followed by beer (0.98), and wine (1.16), and all are significant. All goods are normal (i.e. the expenditure elasticity is positive) and reflect that if total household expenditure increases by 10%, the consumption of distilled spirits, wines, and beers would increase by 7.1%, 11.6% and 9.8%, respectively.

The sensitivity of the quality decision to total expenditure is reported in the quality-expenditure elasticity, where wine is the beverage that reports the greatest sensitivity, with a quality-expenditure elasticity of 0.20, followed by distilled beverages (0.15) and lastly beers (0.12). Thus, if total household expenditure increases by 10%, the chosen unit value (proxy for quality) of distilled beverages, wines, and bears increases by an average of 1.5%, 2% and 1.2%, respectively.

The estimations by socio-demographic groups (not shown) suggest that households from the Metropolitan Area of Santiago, with male heads of the household and with incomplete secondary education have more elastic spirit elasticities than households from other regions, households with female heads, and those with heads who completed secondary education. No difference in the other beverages and elasticities was found. However, in all the groups which were less sensible to changes in prices (more inelastic) the elasticity of spirits was not significant.

As explained above, the sample has a large proportion of households with errors in the unit of measurement of alcohol purchases, which were imputed using hot-deck imputation. To check for robustness, we estimated elasticities only for the sample with no errors in the unit of measurement. These results (not shown but available from the authors) show no significant difference from those in Table 5, except for the spirits own-price elasticity, which is non-significant.

## Discussion

The first thing that draws attention in the results is their misalignment with studies on other countries. While the literature finds that distilled beverages have greater elasticity than wines and beers (-0.8 for distilled spirits (7)), this study found distilled beverages to be the least elastic (-0.14) and, beers, were shown to be the least elastic [9].

It should be recalled that the aforementioned studies report average elasticities, obtained by taking numerous individual studies with a significant difference in results among them into consideration. In one of them, for example, the elasticities reported by different studies on an aggregate level range between -0.8 and -2 for distilled spirits; -0.64 and -1 for wines and -0.25 and 0.24 for beers [22].

The differences in the estimated price elasticities for different countries were addressed in another meta-analysis that included 50 estimates for distilled spirits, 54 for wines and 46 for beers [23]. The minimum elasticities (in absolute value) reported by those studies were 0.1, 0.05, and 0.09 for distilled spirits, wines and beers, respectively. The maximum elasticities reported for the same group of beverages were (in absolute value) 2, 1.8, and 1.2, respectively. This study concludes that the differences could be explained by the per capita consumption and market share of each beverage, where a higher per capita consumption or market share was related to more inelastic price elasticities.

In the case of Chile (or other wine-producing countries, such as France and Italy), distribution of pure alcohol consumption is heavily concentrated in distilled spirits and wines (over 70% of pure alcohol consumed) [1]. In other countries like the United States and Canada, consumption of pure alcohol is mainly concentrated in beers (around 50%). Thus, the lower price elasticities for distilled spirits and its higher consumption share (with wines) could be due to the particular market situation of these beverages in Chile. Chile has comparative advantages in pisco production (the national distilled beverage) and is one of the world's largest producers of wine (seventh in 2014 [24]). This means that, compared to international levels, the average price of spirits is lower than the average price of beers. In addition, pisco and rum (the other spirit beverage consumed) are usually used as inputs for cocktails and combined with other beverages. This means that the quantity of spirits consumed with each drink is relatively small, decreasing the impact that a price increase would have on the total cost of the drink, and reducing the price elasticity of spirits (in absolute value).

Table 6 shows the price of distilled spirits and wines compared to beers for some of the countries in the region and for high-income countries that are also important producers of alcoholic beverages. In Chile, one can see that the relative price of distilled spirits as well as wines is significantly lower than in the countries that are mostly considered in elasticity studies (e.g., USA, Canada, UK, China). In Chile the comparative price of wines and distilled spirits compared to beers is fairly low.

**Table 6:** Relative price of spirits and wine compared to beer in 2013 (in USD 2016 constant prices).

Geographies	Spirits	Wine
Spain	10.0	1.2
Italy	12.0	1.2
**Chile**	**7.5**	**1.4**
Portugal	9.1	1.5
Argentina	11.3	1.5
Uruguay	7.2	1.6
Germany	10.8	2.1
Ireland	9.0	2.2
France	9.2	2.3
Greece	13.3	2.3
United Kingdom	7.8	2.4
Peru	10.1	2.8
Canada	8.7	3.1
USA	8.5	3.3
Brazil	4.9	4.2
Colombia	7.6	4.9
Mexico	11.1	6.7
Ecuador	6.8	9.8

Source: Euromonitor International.

It is highly likely that wine-producing or spirit-producing countries not only have lower prices (before tax) in the national market, but also that the dispersion of these prices will be relatively high. In these cases, when faced with an increase in the price of spirits or wines, consumers have a wide variety of brands at lower prices that they can switch to (down-trade), reducing the effect on total amounts consumed (and, therefore, reducing the price-elasticity of wine, though increasing the price-elasticity of each of the brands or varieties of wine that are sold). This is indirectly proven by the quality elasticities found, where wine quality elasticity almost doubles that of beer, while spirits elasticity is significantly higher than that of beer. This elasticity is ultimately an indication of households' willingness to buy more expensive or cheaper goods (within the same type of beverage) if their purchasing power increases or decreases.

In terms of policies to control alcohol consumption, the results obtained reveal several elements that must be considered. First, all beverages have negative and inelastic price elasticities, which implies that a tax increase in alcoholic beverages will reduce the volumes consumed. Given that these elasticities are less than one (in absolute value), a higher tax will also entail an increase in tax collection. Thus, an increase in the tax on alcoholic beverages will produce two positive effects, reduced consumption of alcoholic beverages (and the social costs incurred from said consumption) [4], and increased revenue collection.

Second, the low relative price of distilled spirits and wines (which have a higher alcohol content than beers) and their low elasticity (possibly due to a wide price variance) makes it necessary for the tax on alcoholic beverages, to be specific and non-ad valorem. While the ad-valorem tax discourages acquisition of more expensive goods (because the tax is a percentage of the value acquired), a specific tax discourages the purchase of quantities of the good. What causes damage to health is the pure alcohol contained in the beverage, and therefore the specific tax ought to be related to alcohol content. Thus, distilled spirits and wines would significantly increase in cost compared to beer, creating a greater disincentive for their consumption. At the same time, among beverages with the same alcohol content, the cheaper brands would experience a higher increase in price, discouraging downtrading.

An alternative to minimize downtrading, which is not mutually exclusive of taxes, is to impose minimum prices (by alcohol content), which has been presented as cost-effective [25] and was recently adopted in Scotland. Unlike Pigouvian taxes such as a specific tax on alcohol, minimum prices do not necessarily correct for externalities and do not generate fiscal revenues. Minimum prices increase revenue for producers when demand is inelastic, and are therefore an inferior policy option to Pigouvian taxes. A specific tax and minimum prices have the disadvantage of being eroded by inflation. However, this would not be a problem in Chile which has a mechanism to automatically update all taxes according to inflation.

Taxes and minimum prices (in addition to other measures such as forbidding "happy hours," discounts on quantity, and promotions) are particularly effective with young people, who are the most susceptible to suffering injury due to excessive consumption of alcoholic beverages [26–29]. According to the available evidence [4, 25, 30], the youngest age groups are the most sensitive to price variations, meaning that any tax increase that is added to the sales price would have a greater differential impact on consumption by young people.

## Limitations

This work's main limitations has to do with the quality of the data available. Unlike other methods, the imputation method used does not reduce the variance in observations, as it replicates the distribution of known observations. However, the actual impact that these imputations may have on the elasticities obtained is unknown.

The large proportion of observations with missing data may be because the dataset used to conduct the estimations was the pre-cleaned data, that is, the version of the dataset before any imputation made by the Statistical Institute. Household Budget Surveys conducted in other developing countries seem to be of better quality, in terms of missing data, only because the version available to the public has already been corrected for missing data (ie, missing data have been imputed). In such cases, the extent of the missing data is unknown and the method for imputing missing data is opaque. In that respect, we find that it is a better option to have a dataset where data problems can be identified and dealt with accordingly.

The quality of the data also made it impossible to estimate the effects of substitute goods or those which complement the consumption of alcoholic beverages, such as tobacco or non-alcoholic beverages, given that records of these goods present even bigger data problems than the data on alcoholic beverages.
